# Synthesis and biological evaluations of oleanolic acid indole derivatives as hyaluronidase inhibitors with enhanced skin permeability

**DOI:** 10.1080/14756366.2021.1956487

**Published:** 2021-07-26

**Authors:** Hao He, Huifang Li, Toyosi Akanji, Shengli Niu, Zhujun Luo, Dongli Li, Navindra P. Seeram, Panpan Wu, Hang Ma

**Affiliations:** a School of Biotechnology and Health Sciences, International Healthcare Innovation Institute (Jiangmen), Wuyi University, Jiangmen, China; b Bioactive Botanical Research Laboratory, Department of Biomedical and Pharmaceutical Sciences, College of Pharmacy, University of Rhode Island, Kingston, RI, USA; c Key Laboratory of Livestock Infectious Diseases in Northeast China, Ministry of Education, College of Animal Science and Veterinary Medicine, Shenyang Agricultural University, Shenyang, China

**Keywords:** Oleanolic acid, indole, hyaluronidase, skin permeability, skin protection

## Abstract

Oleanolic acid (OA) is a natural cosmeceutical compound with various skin beneficial activities including inhibitory effect on hyaluronidase but the anti-hyaluronidase activity and mechanisms of action of its synthetic analogues remain unclear. Herein, a series of OA derivatives were synthesised and evaluated for their inhibitory effects on hyaluronidase. Compared to OA, an induction of fluorinated (**6c**) and chlorinated (**6g**) indole moieties led to enhanced anti-hyaluronidase activity (IC_50_ = 80.3 vs. 9.97 and 9.57 µg/mL, respectively). Furthermore, spectroscopic and computational studies revealed that **6c** and **6g** can bind to hyaluronidase protein and alter its secondary structure leading to reduced enzyme activity. In addition, OA indole derivatives showed feasible skin permeability in a slightly acidic environment (pH = 6.5) and **6c** exerted skin protective effect by reducing cellular reactive oxygen species in human skin keratinocytes. Findings from the current study support that OA indole derivatives are potential cosmeceuticals with anti-hyaluronidase activity.

## Graphical Abstract

**Figure:**
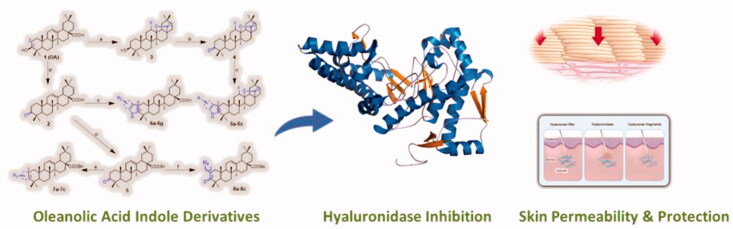

A series of novel oleanolic acid (OA) derivatives are synthesised

OA indole derivatives **6a**-**6g** show potent inhibition on hyaluronidase activity

**6c** and **6g** interacts with hyaluronidase protein to reduce enzyme activity

**6c** and **6g** are skin permeable at pH 6.5 and **6c** shows antioxidant effect in human skin keratinocytes

## Introduction

Oleanolic acid (OA) is a pentacyclic triterpenoid that is ubiquitous in the plant kingdom. It is a bioactive component of numerous botanical extracts that are commonly used as cosmeceutical ingredients in various skincare products. The skin beneficial effects of OA are supported by several biological studies showing that it may exert promising anti-inflammatory, antioxidant, and anti-skin-ageing activities^1–3^. It also has been reported that OA’s anti-skin-ageing effects may be attributed to its inhibitory effect on several skin wrinkle formation related enzymes such as hyaluronidase (HAase)^4^, which is a key enzyme for the metabolism of hyaluronic acid (also known as hyaluronan; HA). HAase catalyses the reaction of degradation of HA to form monosaccharides by cleaving its glycosidic bonds^5^. HAases can be categorised into different types by their origins and mechanism of action. For instance, mammalian produced HAases, also termed as the endo-β-N-acetylhexosaminidases, can break down the β-1,4-glycosidic linkages of HA to form tetrasaccharides, whereas HAases produced by worms (i.e. leech) are endo-β-D-glucuronidases, which catalyses the cleavage of the β-1,3-glycosidic bonds to release pentasaccharides and hexasaccharides^6^. In addition, the types of HAases, including acidic ones (activated in acidic environment; pH of 3 to 4) and neutral ones (biologically functional at pH of 5 to 8), are varied by their catalytic activity in different pH environment^6^. HA is one of the major components that comprise the skin extracellular matrix (ECM) system, which is a three-dimensional network with crucial physiological functions such as maintaining the skin structure and retaining water molecules^7^. Published dermatological studies suggest that abnormal hyperactivity of HAase may lead to exacerbated degradation of skin epidermal HA, which is a histochemical hallmark for aged skin^8^. The HA-HAase homeostasis is a crucial factor that mediates a variety of skin ageing related physiological events including ECM structural integrity, wrinkle formation, and skin moisture retention^9^^,^^10^. Thus, HAase inhibitors are considered as promising cosmeceuticals with potential anti-skin-ageing effects, thereby attracted considerable amount of research interest^11–13^.

Several biochemical based assays have been developed to evaluate the inhibitory effects of small molecules on the activity of HAase. Viscosimetry assays measuring the ‘half viscosity time’ or ‘half time of the substrate’ were first used to evaluate HAase activity^14^. More advanced analytical methods, such as a spectrophotometric HAase assay measuring the undegraded HAase substrate^15^, were developed to accurately assess HAase activity. Moreover, the development of microplate based enzymatic HAase assays enabled the measurement of HAase activity at a larger scale, which led to the discovery of numerous HAase inhibitors^16^^,^^17^. Among these inhibitors, phytochemicals from medicinal plants, including polyphenols, terpenes (including monoterpenes, diterpenes, sesquiterpenes, and triterpenes) and their glycosides, showed promising anti-HAase effects with different mechanisms of action^6^^,^^18^^,^^19^. Notably, pentacyclic triterpenoids, such as OA and ursolic acid (UA), have been identified as active HAase inhibitors and are often used as the positive controls in anti-HAase enzymatic assays^2^^,^^4^. In addition, a series of pentacyclic triterpenoid analogues with an UA skeleton were chemically synthesised and a quantitative structure activity relationship study showed that the modifications (e.g. acetylation and methylation) of UA can result in enhanced inhibitory effects on HAase^20^. Moreover, augmented anti-HAase activity of a group of carboxamide derivatives via the induction of indole moieties has been reported^21^. However, to date, it is unclear whether enhanced anti-HAase activity can be achieved by the induction of indole groups to the OA skeleton.

In light of our laboratory’s research interest to study the potential skin beneficial effects of HAase inhibitors, herein, we aimed to: 1) synthesise a series of OA derivatives with an indole moiety and evaluate their inhibition against bovine testicular HAase; 2) study the leading compounds’ mechanisms of inhibition using biophysical and computational methods; 3) evaluate OA indole derivatives’ cosmeceutical properties including skin permeability and antioxidant activity in human skin cells.

## Materials and methods

### Chemicals and reagents

Hydrogen peroxide (H_2_O_2_) solution, dimethyl sulfoxide (DMSO), bovine serum albumin (BSA), HAase from bovine testes (type IV-S; CAS number: 37326–33-3), and hyaluronic acid from rooster comb (mw from 1000000 to 4000000 Da; CAS Number: 9067–32-7) were purchased from Sigma-Aldrich Chemical Co. (St. Louis, MO, USA). Oleanolic acid (OA), penylhydrazine hydrochloride, aldehydes, selectflour, and potassium hydroxide were obtained from Macklin Co., Ltd. (Shanghai, China; purity > 98%). All other solvents used in this study were purchased from Aladdin Chemical (Shanghai, China). The Jones reagent was prepared with a method as previously reported^22^.

### Spectrometer

^1^H NMR and ^13 ^C NMR spectra were recorded on a Bruker DPX-500 MHz spectrometer (Bruker Biospin, Rheinstetten, German) with chemical shifts (*d*) given in parts per million (ppm) with trimethylsilane (TMS) as an internal standard. High-resolution mass spectra (HRMS) were obtained on a Thermo Scientific Q Exactive UHMR (ultra-high mass range) hybrid quadrupole-orbitrap mass spectrometer (Thermo Fisher Scientific; Waltham, MA, USA).

### General procedure for the preparation of OA analogues

#### Synthesis of oleanolic acid analogue 2

OA (500 mg, 1.1 mmol) was added into acetone (20 mL) and stirred at 0 °C until it was completely dissolved and then the Jones reagent (5 mL) was slowly added into the solution and stirred until the colour of the mixture turned yellow for 5 min. The reaction was monitored by the thin layer chromatography (TLC) and acetone in the reaction mixture was removed under reduced pressure after the reaction completed. The remaining reactants were dissolved in ethyl acetate and washed with deionised water, and then dried over anhydrous magnesium sulphate. Next, the crude product was purified on a silica gel column with petroleum ether/ethyl acetate (v/v = 12:1, R_f_ = 0.3) to obtain the intermediate analogue **2** with a yield of 80.6% (white solid). ^1^H NMR (500 MHz, CDCl_3_) δ 5.23 (t, J = 3.5 Hz, 1H), 2.77 (dd, J = 13.7, 4.1 Hz, 1H), 2.53–2.43 (m, 1H), 2.34–2.26 (m, 1H), 1.96–1.87 (m, 2H), 1.87–1.78 (m, 2H), 1.75–1.61 (m, 3H), 1.60–1.49 (m, 5H), 1.43–1.39 (m, 3H), 1.30–1.23 (m, 4H), 1.19–1.13 (m, 2H), 1.08 (s, 3H), 1.01 (s, 3H), 0.98 (s, 3H), 0.96 (s, 3H), 0.86 (s, 3H), 0.84 (s, 3H), 0.74 (s, 3H). ^13 ^C NMR (151 MHz, CDCl_3_) δ 217.75, 183.75, 143.65, 122.40, 55.31, 47.45, 46.89, 46.58, 45.82, 41.73, 41.04, 39.27, 39.10, 36.81, 34.15, 33.80, 33.06, 32.40, 32.16, 30.68, 27.68, 26.45, 25.84, 23.56, 23.49, 22.91, 21.45, 19.55, 17.01, 15.02. HRMS (ESI-MS) m/z: [M + Na]^+^ calcd for C_30_H_46_NaO_3_: 477.3339; found: 477.3342.

#### Synthesis of oleanolic acid analogue 3

OA (500 mg, 1.1 mmol) and selectfluor (tetrafluoroborate, 3.3 mmol) were dissolved in the mixed solution of dioxane (4 mL) and nitromethane (6 mL), and stirred at 80 °C for 4 h monitored by the TLC. The reaction mixture was concentrated under reduced pressure and then extracted with ethyl acetate. The mixture was then washed with deionised water and dried with anhydrous magnesium sulphate. Next, the crude product was purified on a silica gel column with petroleum ether/ethyl acetate (v/v = 8:1, R_f_=0.3) to obtain the intermediate analogue **3** as white solid with a yield of 50.3%. ^1^H NMR (500 MHz, CDCl_3_) δ 4.58 (dt, *J* = 46.6, 2.6 Hz, 1H), 3.24 (dd, *J* = 11.6, 4.6 Hz, 1H), 2.20–2.07 (m, 2H), 2.03–1.91 (m, 2H), 1.91–1.81 (m, 2H), 1.77–1.70 (m, 2H), 1.70–1.63 (m, 3H), 1.63–1.52 (m, 5H), 1.51–1.35 (m, 3H), 1.35–1.26 (m, 3H), 1.25 (s, 3H), 1.21 (dd, *J* = 14.1, 6.5 Hz, 1H), 1.13 (s, 3H), 1.01 (d, *J* = 2.6 Hz, 6H), 0.92 (s, 3H), 0.89 (s, 3H), 0.79 (s, 3H), 0.78–0.73 (m, 1H). ^13 ^C NMR (151 MHz, CDCl_3_) δ 179.44, 97.37, 96.01,88.04, 78.74, 55.00, 50.87, 44.67, 44.35, 41.93, 41.71f, 38.89, 38.54, 36.41, 34.09, 33.52, 33.22, 31.52, 27.99, 27.47, 27.36, 27.18, 25.73, 23.80, 21.07, 18.32, 18.03, 17.69, 16.07, 15.38. HRMS (ESI-MS) m/z: [M + Na]^+^ calcd for C_30_H_47_FNaO_3_: 497.3401; found: 497.3405.

#### Synthesis of oleanolic acid analogues 4

Analogue **3** (500 mg, 1.1 mmol) in acetone (20 mL) was dissolved and stirred at 0 °C followed by adding the Jones reagent (5 mL) and stirring until the mixture turned yellow for 5 min and monitored by the TLC. The reaction mixture was then removed under reduced pressure and the remainder was dissolved in ethyl acetate and washed with deionised water. After drying the extract over anhydrous magnesium sulphate, it was purified on a silica gel column with petroleum ether/ethyl acetate (v/v = 12:1, R_f_=0.3) to obtain the intermediate **4** as white solid with a yield of 69.6%. ^1^H NMR (500 MHz, CDCl_3_) δ 4.60 (dt, *J* = 46.6, 2.6 Hz, 1H), 2.59–2.50 (m, 1H), 2.47 (tt, *J* = 11.8, 3.6 Hz, 1H), 2.30–2.14 (m, 1H), 2.14–2.09 (m, 1H), 2.09–2.01 (m, 1H), 2.00–1.91 (m, 3H), 1.91–1.79 (m, 2H), 1.78–1.72 (m, 1H), 1.72–1.62 (m, 4H), 1.62–1.53 (m, 2H), 1.53–1.47 (m, 2H), 1.45–1.37 (m, 2H), 1.36–1.31 (m, 2H), 1.27 (s, 3H), 1.24 (dd, *J* = 9.3, 4.5 Hz, 1H), 1.18 (s, 2H), 1.12 (s, 3H), 1.06 (s, 3H), 1.04–0.99 (m, 6H), 0.92 (s, 3H). ^13 ^C NMR (151 MHz, CDCl_3_) δ 179.44, 96.69, 88.04, 78.74, 55.00, 50.87, 44.67, 44.35, 41.93, 41.71, 41.70, 38.89, 38.54, 36.41, 34.09, 33.52, 33.22, 31.52, 27.99, 27.47, 27.36, 27.18, 25.73, 23.80, 21.07, 18.32, 18.01, 17.82, 16.07, 15.38. HRMS (ESI-MS) m/z: [M + Na]^+^ calcd for C_30_H_45_FNaO_3_: 495.3245; found: 495.3245.

#### Synthesis of oleanolic acid analogues 5a-5c

Analogue **4** (0.2 mmol) and substituted phenylhydrazines (0.4 mmol) were dissolved in acetic acid with refluxing at 110 °C overnight. The reaction was monitored by TLC and the reaction mixture was concentrated under reduced pressure. The remainder was dissolved in ethyl acetate and the organic layer was washed with deionised water, and then dried over anhydrous magnesium sulphate. The crude product was purified on a silica gel column with petroleum ether/ethyl acetate (v/v = 20:1–36:1) to obtain intermediate analogues **5a**-**5c**.

Analogue **5a**: followed by aforementioned general procedures, **5a** was prepared *via* the Fischer indole synthesis of **4** with 4-(trifluoromethoxy) phenylhydrazine hydrochloride and purified with petroleum ether/ethyl acetate (v/v = 20:1, R_f_ = 0.50); light yellow solid; yield: 53.2%. ^1^H NMR (500 MHz, CDCl_3_) δ 4.58 (dt, *J* = 46.6, 2.6 Hz, 1H), 3.24 (dd, *J* = 11.6, 4.6 Hz, 1H), 2.20–2.07 (m, 2H), 2.03–1.91 (m, 2H), 1.91–1.81 (m, 2H), 1.77–1.70 (m, 2H), 1.70–1.63 (m, 3H), 1.63–1.52 (m, 5H), 1.51–1.35 (m, 3H), 1.35–1.26 (m, 3H), 1.25 (s, 3H), 1.21 (dd, *J* = 14.1, 6.5 Hz, 1H), 1.13 (s, 3H), 1.01 (d, *J* = 2.6 Hz, 6H), 0.92 (s, 3H), 0.89 (s, 3H), 0.79 (s, 3H), 0.78–0.73 (m, 1H). ^13 ^C NMR (151 MHz, CDCl_3_) δ 179.47, 142.89, 142.63, 134.41, 128.40, 120.85, 114.93, 110.78, 110.69, 107.13, 96.57, 88.13, 52.91, 51.05, 44.44, 43.43, 41.91, 38.69, 37.59, 36.94, 34.30, 34.14, 33.23, 32.82, 31.57, 30.86, 29.73, 27.62, 27.40, 26.12, 23.78, 23.18, 21.13, 18.58, 17.82, 17.77, 16.40. HRMS (ESI-MS) m/z: [M + H]^+^ calcd for C_37_H_48_F_4_NO_3_: 630.3565; found: 630.3567.

Analogue **5b**: followed by aforementioned general procedures, **5b** was prepared *via* the Fischer indole synthesis of **4** with 4-fluorophenylhydrazine hydrochloride and purified with petroleum ether/ethyl acetate (v/v = 20:1, R_f_ = 0.35); light yellow solid; yield: 64.3%. ^1^H NMR (500 MHz, CDCl_3_) δ 7.95 (s, 1H), 7.23 (d, *J* = 7.8 Hz, 1H), 7.00 (td, *J* = 7.9, 4.8 Hz, 1H), 6.87 (ddd, *J* = 11.3, 7.8, 0.6 Hz, 1H), 4.69 (dt, *J* = 46.8, 2.4 Hz, 1H), 2.89 (t, *J* = 14.6 Hz, 1H), 2.25 (d, *J* = 15.2 Hz, 1H), 2.23–2.09 (m, 3H), 2.09–1.97 (m, 2H), 1.97–1.88 (m, 2H), 1.80 (dd, *J* = 13.0, 1.7 Hz, 1H), 1.72–1.64 (m, 5H), 1.49–1.40 (m, 3H), 1.36 (s, 3H), 1.33–1.31 (m, 4H), 1.28 (s, 2H), 1.26 (s, 3H), 1.24 (s, 3H), 1.03 (s, 3H), 0.96 (s, 3H), 0.94 (s, 3H). ^13 ^C NMR (151 MHz, CDCl_3_) δ 179.44, 149.30, 141.58, 131.91, 124.01, 119.23, 113.82, 107.40, 106.25, 96.63, 88.11, 52.96, 51.05, 44.44, 43.44, 41.95, 41.85, 38.71, 37.61, 37.20, 34.29, 34.13, 33.24, 32.84, 31.57, 30.89, 29.73, 27.62, 27.40, 26.12, 23.79, 23.20, 21.13, 18.58, 17.82, 17.81, 16.39. HRMS (ESI-MS) m/z: [M + H]^+^ calcd for C_36_H_48_F_2_NO_2_: 564.3648; found: 564.3651.

Analogue **5c**: followed by aforementioned general procedures, **5c** was prepared *via* the Fischer indole synthesis of **4** with 2-chlorophenylhydrazine hydrochloride and purified with petroleum ether/ethyl acetate (v/v = 32:1, R_f_ = 0.49); light yellow solid; yield: 57.3%. ^1^H NMR (500 MHz, CDCl_3_) δ 7.93 (s, 1H), 7.37 (d, *J* = 7.7 Hz, 1H), 7.14 (dd, *J* = 7.6, 0.8 Hz, 1H), 7.03 (t, *J* = 7.7 Hz, 1H), 4.69 (dt, *J* = 46.3, 2.4 Hz, 1H), 2.86 (d, *J* = 14.9 Hz, 1H), 2.25 (d, *J* = 15.0 Hz, 1H), 2.23–2.14 (m, 2H), 2.14–2.04 (m, 2H), 2.04–1.88 (m, 3H), 1.83–1.77 (m, 1H), 1.72–1.61 (m, 5H), 1.49–1.39 (m, 3H), 1.38–1.34 (m, 4H), 1.32 (s, 3H), 1.30–1.26 (m, 5H), 1.24 (s, 3H), 1.03 (s, 3H), 0.96 (s, 3H), 0.94 (s, 3H). ^13 ^C NMR (151 MHz, CDCl_3_) δ 179.55, 141.69, 133.38, 129.78, 120.65, 119.94, 116.76, 116.08, 107.86, 96.75, 88.22, 53.08, 51.17, 44.55, 43.55, 42.06, 41.99, 38.83, 37.70, 37.29, 34.41, 34.25, 33.35, 32.95, 31.69, 30.99, 27.73, 27.51, 26.24, 23.90, 23.29, 21.24, 18.70, 17.935, 17.930, 16.49. HRMS (ESI-MS) m/z: [M + H]^+^ calcd for C_36_H_48_FClNO_2_: 580.3352; found: 580.3354.

#### Synthesis of oleanolic acid analogues 6a-6g

The indole ring substituted analogues **6a**-**6g** were synthesised by employing substituted phenylhydrazine in the Fischer indolization. Analogue **2** (0.2 mmol) and substituted phenylhydrazines (0.4 mmol) were dissolved in acetic acid with refluxing at 110 °C overnight. The reaction was monitored by the TLC. After the reaction completed, acetic acid in the reaction mixture was removed under reduced pressure followed by dissolving the remainder in ethyl acetate. The organic layer was washed with deionised water and dried over anhydrous magnesium sulphate. The crude product was separated by preparative silica gel chromatography in petroleum ether/ethyl acetate (7:1 to 12:1).

Analogue **6a**: followed by aforementioned general procedures, **6a** was prepared *via* the Fischer indole synthesis of **2** with 2-nitrophenylhydrazine hydrochloride and purified with petroleum ether/ethyl acetate (v/v = 8:1, R_f_ = 0.33); light red solid; yield: 30.2%. ^1^H NMR (500 MHz, CDCl_3_) δ 8.39 (d, *J* = 2.2 Hz, 1H), 8.25 (s, 1H), 8.03 (dd, *J* = 8.9, 2.3 Hz, 1H), 7.29 (d, *J* = 8.9 Hz, 1H), 5.41 (t, *J* = 3.6 Hz, 1H), 2.90 (dd, *J* = 13.0, 3.9 Hz, 1H), 2.80 (d, *J* = 15.1 Hz, 1H), 2.22 (d, *J* = 15.2 Hz, 1H), 2.14–2.08 (m, 2H), 2.01 (td, *J* = 13.6, 4.1 Hz, 1H), 1.82 (ddd, *J* = 26.9, 12.3, 5.9 Hz, 3H), 1.71–1.59 (m, 2H), 1.63–1.40 (m, 4H), 1.39–1.32 (m, 1H), 1.32 (s, 3H), 1.30–1.21 (m, 3H), 1.19 (d, *J* = 2.3 Hz, 6H), 1.18–1.13 (m, 1H), 0.96 (s, 3H), 0.94 (s, 3H), 0.92 (s, 3H), 0.86 (s, 3H), 0.92–0.81 (m, 1H). ^13 ^C NMR (151 MHz, CDCl_3_) δ 184.25, 144.38, 143.38, 141.22, 139.40, 127.82, 122.73, 116.97, 115.34, 110.13, 109.51, 53.03, 46.67, 46.37, 45.80, 41.80, 41.12, 39.43, 38.11, 36.40, 34.12, 33.85, 33.10, 32.48, 32.06, 30.73, 30.70, 27.74, 25.82, 23.57, 23.41, 23.13, 22.92, 19.25, 16.82, 15.55. HRMS spectrum of (compound 6a). HRMS (ESI-MS) m/z: [M-H]^-^ calcd for C_36_H_47_N_2_O_4_: 571.3541; found: 571.3514.

Analogue **6b**: followed by aforementioned general procedures, **6b** was prepared *via* the Fischer indole synthesis of **2** with 2-fluorophenylhydrazine and purified with petroleum ether/ethyl acetate (v/v = 7:1, R_f_ = 0.35); light yellow solid; yield: 59.1%. ^1^H NMR (500 MHz, CDCl_3_) δ 7.88 (s, 1H), 7.19 (d, *J* = 7.8 Hz, 1H), 6.97 (td, *J* = 7.8, 4.6 Hz, 1H), 6.83 (dd, *J* = 11.2, 7.8 Hz, 1H), 5.57–5.29 (m, 1H), 2.94–2.85 (m, 1H), 2.76 (d, *J* = 14.9 Hz, 1H), 2.20 (d, *J* = 15.1 Hz, 1H), 2.14–2.08 (m, 2H), 2.01 (td, *J* = 13.6, 4.0 Hz, 1H), 1.88–1.73 (m, 3H), 1.71–1.48 (m, 5H), 1.46–1.32 (m, 3H), 1.31 (s, 3H), 1.29–1.20 (m, 3H), 1.19 (s, 6H), 1.16–1.11 (m, 1H), 0.96 (d, *J* = 2.2 Hz, 6H), 0.92 (s, 3H), 0.86 (s, 3H). ^13 ^C NMR (151 MHz, CDCl_3_) δ 184.30, 150.29, 143.42, 141.75, 132.04, 124.04, 122.83, 119.17, 113.80, 107.82, 106.20, 53.14, 46.70, 46.39, 45.87, 41.82, 41.13, 39.43, 38.15, 36.87, 34.07, 33.85, 33.10, 32.48, 32.13, 30.96, 30.72, 27.76, 25.82, 23.59, 23.45, 23.32, 22.96, 19.25, 16.90, 15.54. HRMS (ESI-MS) m/z: [M-H]^-^ calcd for C_36_H_47_FNO_2_: 544.3596; found: 544.3573.

Analogue **6c**: followed by aforementioned general procedures, **6c** was prepared *via* the Fischer indole synthesis of **2** with 2,4-difluorophenylhydrazine hydrochloride and purified with petroleum ether/ethyl acetate (v/v = 8:1, R_f_ = 0.35); white solid; yield: 53.9%. ^1^H NMR (500 MHz, CDCl_3_) δ 7.83 (d, *J* = 10.7 Hz, 1H), 6.89 (dd, *J* = 9.0, 2.2 Hz, 1H), 6.67 (ddd, *J* = 11.4, 9.5, 2.2 Hz, 1H), 5.55–5.30 (m, 1H), 2.90 (dd, *J* = 14.0, 4.6 Hz, 1H), 2.69 (d, *J* = 15.0 Hz, 1H), 2.18 (d, *J* = 15.2 Hz, 1H), 2.14–2.07 (m, 2H), 2.03 (td, *J* = 13.6, 4.1 Hz, 1H), 1.81 (ddt, *J* = 24.6, 17.7, 10.4 Hz, 3H), 1.72–1.49 (m, 6H), 1.46–1.35 (m, 3H), 1.33 (d, *J* = 6.7 Hz, 3H), 1.30–1.22 (m, 3H), 1.21 (d, *J* = 3.3 Hz, 6H), 0.99–0.91 (m, 9H), 0.88 (s, 3H). ^13 ^C NMR (151 MHz, CDCl_3_) δ 184.71, 157.58, 155.71, 149.09, 147.14, 143.43, 130.87, 122.76, 120.51, 108.18, 98.88, 95.89, 53.07, 46.71, 46.37, 45.86, 41.81, 41.10, 39.42, 38.14, 36.74, 34.18, 33.86, 33.11, 32.49, 32.10, 30.89, 30.72, 27.76, 25.81, 23.59, 23.42, 23.32, 22.93, 19.24, 16.89, 15.52. HRMS (ESI-MS) m/z: [M-H]^-^ calcd for C_36_H_46_F_2_NO_2_: 562.3502; found: 562.3478.

Analogue **6d**: followed by aforementioned general procedures, analogue **6d** was prepared *via* the Fischer indole synthesis of **2** with 2,4-dimethylphenylhydrazine hydrochloride and purified with petroleum ether/ethyl acetate (v/v = 12:1, R_f_ = 0.30); white solid; yield: 71.1%. ^1^H NMR (500 MHz, CDCl_3_) δ 7.45 (s, 1H), 7.08 (s, 1H), 6.77 (s, 1H), 5.40 (d, *J* = 3.6 Hz, 1H), 2.89 (dd, *J* = 14.1, 4.5 Hz, 1H), 2.73 (d, *J* = 14.9 Hz, 1H), 2.43 (d, *J* = 16.0 Hz, 6H), 2.17 (d, *J* = 14.9 Hz, 1H), 2.14–2.06 (m, 2H), 2.01 (td, *J* = 13.5, 3.8 Hz, 1H), 1.87–1.72 (m, 3H), 1.72–1.45 (m, 5H), 1.45–1.36 (m, 3H), 1.28 (d, *J* = 16.2 Hz, 7H), 1.18 (d, *J* = 7.6 Hz, 6H), 1.01–0.90 (m, 9H), 0.86 (s, 3H). ^13 ^C NMR (151 MHz, CDCl_3_) δ 184.00, 143.35, 140.75, 133.83, 128.42, 128.05, 123.36, 122.97, 119.13, 115.46, 107.01, 53.27, 46.69, 46.41, 45.88, 41.83, 41.14, 39.43, 38.11, 36.91, 34.03, 33.87, 33.11, 32.51, 32.18, 31.03, 30.73, 27.77, 25.84, 23.59, 23.46, 23.37, 22.98, 21.41, 19.30, 16.91, 16.71, 15.51. HRMS (ESI-MS) m/z: [M-H]^-^ calcd for C_38_H_52_NO_2_: 554.4004; found: 544.3978.

Analogue **6e**: followed by aforementioned general procedures, **6e** was prepared *via* the Fischer indole synthesis of **2** with 4-bromophenylhydrazine hydrochloride and purified with petroleum ether/ethyl acetate (v/v = 8:1, R_f_ = 0.35); white solid; yield: 40.8%. ^1^H NMR (500 MHz, CDCl_3_) δ 7.76 (s, 1H), 7.54 (d, *J* = 1.9 Hz, 1H), 7.18 (dd, *J* = 8.5, 1.8 Hz, 1H), 5.40 (t, *J* = 3.6 Hz, 1H), 2.96–2.83 (m, 1H), 2.70 (d, *J* = 14.9 Hz, 1H), 2.15 (d, *J* = 15.0 Hz, 1H), 2.13–2.04 (m, 2H), 2.01 (td, *J* = 13.6, 2.9 Hz, 1H), 1.88–1.67 (m, 3H), 1.68–1.46 (m, 5H), 1.44–1.34 (m, 2H), 1.30–1.21 (m, 9H), 1.18 (d, *J* = 9.4 Hz, 6H), 0.95 (s, 3H), 0.92 (s, 6H), 0.85 (s, 3H). ^13 ^C NMR (151 MHz, CDCl_3_) δ 184.37, 143.27, 141.53, 134.52, 129.33, 123.17, 122.62, 119.97, 117.01, 108.14, 104.02, 52.95, 46.52, 46.20, 45.68, 41.63, 40.91, 39.24, 37.93, 36.69, 33.90, 33.67, 32.94, 32.31, 31.93, 30.76, 30.55, 27.57, 25.65, 23.42, 23.26, 23.14, 22.76, 19.06, 16.74, 15.35. HRMS (ESI-MS) m/z: [M-H]^-^ calcd for C_36_H_47_BrNO_2_: 604.2796; found: 604.2770.

Analogue **6f**: followed by aforementioned general procedures, **6f** was prepared *via* the Fischer indole synthesis of **2** with 2-bromophenylhydrazine hydrochloride and purified with petroleum ether/ethyl acetate (v/v = 8:1, R_f_ = 0.35); white solid; yield: 47.7%. ^1^H NMR (500 MHz, CDCl_3_) δ 7.83 (s, 1H), 7.38 (d, *J* = 7.7 Hz, 1H), 6.97 (t, *J* = 7.7 Hz, 1H), 5.42 (t, *J* = 3.7 Hz, 1H), 2.90 (dd, *J* = 13.8, 4.7 Hz, 1H), 2.76 (d, *J* = 15.0 Hz, 1H), 2.22 (d, *J* = 15.1 Hz, 1H), 2.16–2.10 (m, 2H), 2.06–1.98 (m, 1H), 1.90–1.74 (m, 3H), 1.72–1.50 (m, 6H), 1.43 (dt, *J* = 11.4, 2.4 Hz, 2H), 1.34 (s, 3H), 1.28 (t, *J* = 3.6 Hz, 2H), 1.26–1.16 (m, 9H), 0.97 (d, *J* = 3.3 Hz, 6H), 0.94 (s, 3H), 0.88 (s, 3H). ^13 ^C NMR (151 MHz, CDCl_3_) δ 184.55, 143.45, 141.71, 134.70, 129.51, 123.35, 122.80, 120.15, 117.19, 108.32, 104.21, 53.13, 46.70, 46.38, 45.86, 41.81, 41.09, 39.42, 38.11, 36.87, 34.08, 33.85, 33.12, 32.49, 32.11, 30.95, 30.73, 27.75, 25.83, 23.60, 23.45, 23.32, 22.94, 19.24, 16.92, 15.54. HRMS (ESI-MS) m/z: [M-H]^-^ calcd for C_36_H_47_BrNO2: 604.2796; found: 604.2772.

Analogue **6g**: followed by aforementioned general procedures, **6g** was prepared *via* the Fischer indole synthesis of **2** with 4-chlorophenylhydrazine hydrochloride and purified with petroleum ether/ethyl acetate (v/v = 7:1, R_f_ = 0.30); white solid; yield: 42.3%. ^1^H NMR (500 MHz, CDCl_3_) δ 7.86 (s, 1H), 7.32 (d, *J* = 7.8 Hz, 1H), 7.11 (dd, *J* = 7.6, 1.0 Hz, 1H), 6.99 (t, *J* = 7.7 Hz, 1H), 5.39 (t, *J* = 3.7 Hz, 1H), 2.88 (dd, *J* = 13.9, 4.6 Hz, 1H), 2.75 (d, *J* = 15.0 Hz, 1H), 2.20 (d, *J* = 15.1 Hz, 1H), 2.15–2.07 (m, 2H), 2.01 (td, *J* = 13.6, 4.0 Hz, 1H), 1.87–1.75 (m, 3H), 1.68–1.47 (m, 6H), 1.45–1.39 (m, 2H), 1.32 (s, 3H), 1.26–1.25 (m, 1H), 1.24–1.12 (m, 9H), 0.95 (d, *J* = 2.8 Hz, 6H), 0.92 (s, 3H), 0.86 (s, 3H). ^13 ^C NMR (151 MHz, CDCl_3_) δ 183.86, 143.45, 141.76, 133.25, 129.76, 122.80, 120.42, 119.74, 116.60, 115.95, 108.16, 53.15, 46.67, 46.38, 45.88, 41.83, 41.14, 39.43, 38.12, 36.84, 34.08, 33.85, 33.10, 32.48, 32.13, 30.95, 30.72, 27.76, 25.81, 23.59, 23.45, 23.31, 22.97, 19.25, 16.91, 15.53. HRMS (ESI-MS) m/z: [M-H]^-^ calcd for C_36_H_47_ClNO_2_: 560.3301; found: 560.3276.

#### Synthesis of oleanolic acid analogues 7a-7c

The pyridine ring substituted analogues **7a**-**7c** were synthesised by employing the *o*-amino benzaldehyde and *o*-amino pyridylaldehyde in the Claisen Schmidt condensation. Analogue **2** (0.2 mmol) and *o*-amino benzaldehyde or *o*-amino pyridylaldehyde (0.4 mmol) were dissolved in saturated potassium hydroxide alcohol solution with refluxing at 90 °C for 5 h monitored by the TLC. After the reaction completed, alcohol in the reaction mixture was removed under reduced pressure followed by dissolving the remainder in ethyl acetate. The organic layer was washed with deionised water, and dried over anhydrous magnesium sulphate. The crude product was separated by preparative silica gel chromatography in petroleum ether/ethyl acetate (v/v 6:1 to 8:1).

Analogue **7a**: followed by aforementioned general procedures, **7a** was prepared *via* the Claisen Schmidt condensation of **2** with m-amino benzaldehyde and purified with petroleum ether/ethyl acetate (v/v = 6:1, R_f_ = 0.44); white solid; yield: 80.3%. ^1^H NMR (500 MHz, CDCl_3_) δ 8.00 (d, *J* = 7.6 Hz, 1H), 7.75–7.64 (m, 2H), 7.59 (t, *J* = 7.3 Hz, 1H), 7.41 (t, *J* = 7.2 Hz, 1H), 7.36 (d, *J* = 4.3 Hz, 4H), 7.34–7.29 (m, 1H), 5.40 (t, *J* = 3.4 Hz, 1H), 5.10 (q, *J* = 12.6 Hz, 2H), 3.03–2.89 (m, 2H), 2.54 (d, *J* = 15.4 Hz, 1H), 2.05–1.98 (m, 2H), 1.77–1.68 (m, 5H), 1.67–1.62 (m, 2H), 1.61–1.48 (m, 5H), 1.45–1.41 (m, 5H), 1.41–1.35 (m, 2H), 1.24–1.21 (m, 2H), 1.20 (s, 3H), 1.16–1.12 (m, 1H), 0.95 (s, 3H), 0.92 (s, 3H), 0.86 (s, 3H), 0.72 (s, 3H). ^13 ^C NMR (151 MHz, CDCl_3_) δ 177.48, 166.06, 147.40, 143.79, 136.44, 135.27, 128.90, 128.68, 128.45, 128.45, 128.06, 128.06, 128.01, 127.97,500 0.92, 126.55, 125.39, 122.50, 66.00, 53.95, 46.88, 46.02, 45.90, 45.59, 42.00, 41.64, 40.25, 39.26, 36.24, 33.93, 33.14, 32.42, 32.39, 32.30, 30.75, 27.66, 25.74, 25.35, 23.66, 23.52, 23.15, 20.49, 16.71, 15.04. HRMS (ESI-MS) m/z: [M + H]^+^ calcd for C_44_H_56_NO_2_: 630.43056; found: 630.42859.

Analogue **7b**: followed by aforementioned general procedures, **7b** was prepared *via* the Claisen Schmidt condensation of **2** with 2-amino-3-pyridinecarboxaldehyde and purified with petroleum ether/ethyl acetate (v/v = 8:1, R_f_ = 0.44); white solid; yield: 66.0%. ^1^H NMR (600 MHz, CDCl_3_) δ 9.01 (dd, *J* = 4.2, 1.9 Hz, 1H), 8.07 (dd, *J* = 8.1, 1.9 Hz, 1H), 7.75 (s, 1H), 7.38 (dd, *J* = 8.1, 4.2 Hz, 1H), 7.35 (d, *J* = 4.4 Hz, 3H), 7.33–7.29 (m, 1H), 5.39 (t, *J* = 3.4 Hz, 1H), 5.09 (q, *J* = 12.6 Hz, 2H), 3.00–2.93 (m, 2H), 2.55 (d, *J* = 15.5 Hz, 1H), 2.12 (s, 1H), 2.06–1.96 (m, 3H), 1.78–1.63 (m, 6H), 1.62–1.53 (m, 3H), 1.53–1.50 (m, 2H), 1.49 (s, 3H), 1.47 (s, 3H), 1.42–1.32 (m, 2H), 1.28–1.23 (m, 1H), 1.19 (s, 3H), 1.14 (dd, *J* = 10.5, 3.0 Hz, 1H), 0.94 (s, 3H), 0.91 (s, 3H), 0.86 (s, 3H), 0.71 (s, 3H). ^13 ^C NMR (151 MHz, CDCl_3_) δ 177.45, 170.37, 155.01, 152.20, 143.89, 136.42, 136.24, 136.13, 130.41, 128.45, 128.45, 128.06, 128.06, 127.97, 122.33, 122.14, 121.23, 66.01, 53.68, 46.86, 45.90, 45.72, 45.56, 42.00, 41.64, 40.77, 39.25, 36.23, 33.91, 33.14, 32.39, 32.36, 32.24, 30.75, 27.64, 25.73, 25.38, 23.65, 23.51, 23.13, 20.46, 16.68, 15.08. HRMS (ESI-MS) m/z: [M + H]^+^ calcd for C_43_H_55_N_2_O_2_: 631.42581; found: 631.42365.

Analogue **7c**: followed by aforementioned general procedures, **7c** was prepared *via* the Claisen Schmidt condensation of **2** with 4-amino-3-pyridinecarboxaldehyde and purified with petroleum ether/ethyl acetate (v/v = 8:1, R_f_ = 0.4); white solid; yield: 56.0%. ^1^H NMR (500 MHz, CDCl_3_) δ 9.12 (s, 1H), 8.62 (d, *J* = 5.9 Hz, 1H), 7.83 (s, 1H), 7.81 (d, *J* = 5.9 Hz, 1H), 7.36 (s, 2H), 7.35 (s, 2H), 7.34–7.29 (m, 1H), 5.39 (t, *J* = 3.5 Hz, 1H), 5.09 (q, *J* = 12.6 Hz, 2H), 3.03–2.91 (m, 2H), 2.56 (d, *J* = 15.5 Hz, 1H), 2.09–2.05 (m, 1H), 2.04–1.97 (m, 2H), 1.78–1.72 (m, 2H), 1.72–1.61 (m, 5H), 1.60–1.47 (m, 5H), 1.43 (s, 3H), 1.42 (s, 3H), 1.41–1.34 (m, 2H), 1.19 (s, 3H), 1.16–1.10 (m, 1H), 0.94 (s, 3H), 0.92 (s, 3H), 0.85 (s, 3H), 0.71 (s, 3H). ^13 ^C NMR (151 MHz, CDCl_3_) δ 177.45, 171.86, 151.59, 149.40, 145.20, 143.85, 136.42, 134.81, 130.79, 128.45, 128.45, 128.05, 128.05, 127.97, 122.63, 122.32, 121.78, 66.00, 53.75, 46.86, 46.05, 45.89, 45.53, 41.99, 41.62, 40.91, 39.24, 36.15, 33.91, 33.13, 32.38, 32.36, 32.22, 30.75, 27.64, 25.73, 25.39, 23.65, 23.50, 23.13, 20.47, 16.69, 15.07. HRMS (ESI-MS) m/z: [M + H]^+^ calcd for C_43_H_55_N_2_O_2_: 631.42581; found: 631.42407.

#### Synthesis of oleanolic acid analogues 8a-8c

The analogues **8a**-**8c** were synthesised by employing substituted 2-nitrobenzaldehyde in the Claisen Schmidt Condensation. Analogue **2** (0.2 mmol) and 2-nitrobenzaldehyde (0.4 mmol) were dissolved in saturated potassium hydroxide alcohol solution at room temperature for 10 h monitored by the TLC. After the reaction completed, alcohol in the reaction mixture was removed under reduced pressure followed by dissolving the remainder in ethyl acetate. The organic layer was washed with deionised water, and dried over anhydrous magnesium sulphate and. The crude product was separated by preparative silica gel chromatography in petroleum ether/ethyl acetate (v/v 6:1 to 8:1).

Analogue **8a**: followed by aforementioned general procedures, **8a** was prepared *via* the Claisen Schmidt condensation of **2** with 5-hydroxy-2-nitrobenzaldehydeand purified with petroleum ether/ethyl acetate (v/v = 8:1, R_f_ = 0.3); White solid; Yield: 52.5%. ^1^H NMR (500 MHz, CDCl_3_) δ 8.15 (d, *J* = 9.1 Hz, 1H), 7.63 (s, 1H), 7.36–7.29 (m, 5H), 6.92 (dd, *J* = 9.1, 2.5 Hz, 1H), 6.63 (d, *J* = 2.4 Hz, 1H), 5.20 (t, *J* = 3.3 Hz, 1H), 5.13–5.03 (m, 2H), 2.86 (dd, *J* = 13.7, 3.9 Hz, 1H), 2.62 (d, *J* = 15.5 Hz, 1H), 2.02–1.87 (m, 3H), 1.69 (ddt, *J* = 31.3, 27.5, 8.4 Hz, 6H), 1.60–1.53 (m, 3H), 1.49–1.39 (m, 4H), 1.36–1.31 (m, 2H), 1.20 (s, 3H), 1.16 (s, 3H), 1.11 (d, *J* = 5.8 Hz, 3H), 1.11–1.05 (m, 2H), 0.89 (s, 3H), 0.87 (s, 3H), 0.84 (s, 3H), 0.61 (s, 3H). ^13 ^C NMR (151 MHz, CDCl_3_) δ 209.33, 178.17, 160.96, 143.67, 140.49, 136.11, 136.01, 134.96, 134.89, 128.51, 128.51, 128.14, 128.09, 128.05, 127.67, 122.10, 116.87, 115.92, 66.37, 53.68, 46.94, 46.25, 45.79, 45.46, 42.98, 42.02, 41.68, 39.24, 36.88, 33.84, 33.07, 32.30, 32.04, 30.71, 28.66, 27.58, 25.69, 23.64, 23.36, 23.06, 22.85, 20.06, 16.58, 15.22. HRMS (ESI-MS) m/z: [M-H]^-^ calcd for C_44_H_54_NO_6_: 692.39566; found: 692.39282.

Analogue **8 b**: followed by aforementioned general procedures, **8 b** was prepared *via* the Claisen Schmidt condensation of **2** with 5-methoxy-2-nitrobenzaldehydeand purified with petroleum ether/ethyl acetate (v/v = 8:1, R_f_ = 0.32); white solid; yield: 59.0%. ^1^H NMR (500 MHz, CDCl_3_) δ 8.20 (d, *J* = 9.2 Hz, 1H), 7.63 (d, *J* = 2.1 Hz, 1H), 7.35–7.28 (m, 5H), 6.93 (dd, *J* = 9.2, 2.7 Hz, 1H), 6.68 (dd, *J* = 2.9, 0.6 Hz, 1H), 5.24 (t, *J* = 3.5 Hz, 1H), 5.10–5.01 (m, 2H), 3.90 (s, 3H), 2.88 (dd, *J* = 13.8, 4.1 Hz, 1H), 2.59 (d, *J* = 15.6 Hz, 1H), 2.00–1.89 (m, 2H), 1.83–1.76 (m, 1H), 1.73–1.64 (m, 4H), 1.61–1.54 (m, 4H), 1.49–1.39 (m, 4H), 1.35–1.28 (m, 3H), 1.19 (s, 3H), 1.15 (s, 3H), 1.12 (s, 3H), 1.09–1.06 (m, 1H), 0.90 (s, 3H), 0.88 (s, 3H), 0.86 (s, 3H), 0.62 (s, 3H). ^13 ^C NMR (151 MHz, CDCl_3_) δ 207.58, 177.37, 163.08, 143.97, 141.04, 136.35, 135.30, 135.14, 134.83, 128.44, 128.44, 128.05, 128.05, 127.98, 127.65, 121.98, 115.90, 113.29, 66.01, 56.05, 53.59, 46.81, 45.98, 45.89, 45.29, 42.82, 41.98, 41.58, 39.22, 36.78, 33.84, 33.08, 32.29, 31.99, 30.69, 28.85, 27.59, 25.65, 23.62, 23.45, 23.08, 22.76, 20.11, 16.56, 15.19. HRMS (ESI-MS) m/z: [M + Na]^+^ calcd for C_45_H_57_NO_6_Na: 730.40781; found: 730.40540.

Analogue **8c**: followed by aforementioned general procedures, **8c** was prepared *via* the Claisen Schmidt Condensation of **2** with 4-acetamidobenzaldehyde and purified with petroleum ether/ethyl acetate (v/v = 6:1, R_f_ = 0.38); white solid; yield: 34.0%. ^1^H NMR (500 MHz, CDCl_3_) δ 7.55 (d, *J* = 8.4 Hz, 2H), 7.47 (s, 1H), 7.39 (d, *J* = 8.5 Hz, 2H), 7.36–7.32 (m, 4H), 7.32–7.28 (m, 1H), 5.34 (t, *J* = 3.4 Hz, 1H), 5.07 (dd, *J* = 31.4, 12.5 Hz, 2H), 2.98 (d, *J* = 16.3 Hz, 1H), 2.94 (dd, *J* = 13.8, 3.8 Hz, 1H), 2.26 (dd, *J* = 16.2, 2.1 Hz, 1H), 2.20 (s, 3H), 2.03–1.86 (m, 3H), 1.75–1.68 (m, 4H), 1.64–1.55 (m, 3H), 1.49 (dd, *J* = 17.8, 11.4 Hz, 3H), 1.44–1.30 (m, 4H), 1.23–1.20 (m, 2H), 1.19 (s, 3H), 1.16 (s, 3H), 1.13 (s, 3H), 0.93 (s, 3H), 0.91 (s, 3H), 0.82 (s, 3H), 0.65 (s, 3H). ^13 ^C NMR (151 MHz, CDCl_3_) δ 207.87, 177.46, 168.32, 143.81, 138.12, 136.86, 136.36, 132.96, 131.86, 131.43, 131.43, 128.44, 128.44, 128.06, 128.06, 127.98, 122.31, 119.34, 119.34, 66.02, 52.93, 46.87, 45.88, 45.42, 45.12, 44.28, 42.03, 41.66, 39.22, 36.21, 33.89, 33.11, 32.34, 31.93, 30.74, 29.79, 27.61, 25.66, 24.74, 23.70, 23.65, 23.13, 22.66, 20.36, 16.53, 15.28. HRMS (ESI-MS) m/z: [M + Na]^+^ calcd for C_46_H_59_NO_4_Na: 712.43363; found: 712.43085.

### Sample preparation

Given that OA and its derivatives are poorly dissolved in phosphate buffer solution (PBS), we first dissolved test samples in methyl sulfoxide (DMSO) as stock solutions at a concentration of 4 mg/mL. The DMSO stock solution of each sample was diluted with PBS to reach to desired concentrations and the DMSO content in the assay reaction mixture was less than 1%, which did not induce significant inhibition of the enzyme activity (inhibition rate less than 3%).

### Hyaluronidase inhibition assay

Inhibitory effect on bovine testicular HAase activity was evaluated using a reported method with minor modifications^23^. It is noted that long HA chains and HAase may form of non-active complexes at low ionic condition, which can impede the catalytic activity of HAase and disrupt the HAase inhibition assay^24^. This interference can be avoided by adding a positively charged protein to balance the ionic strength of the reaction system and recover the activity of HAase^24^. Therefore, bovine serum albumin (BSA; 0.01%) was added to the reaction buffer (PBS; 20 mM) in acidic condition (pH 3.75), which provides an optimal electrostatic environment for the enzyme reaction. Test samples (5 μL; concentration ranging from 2.5 to 40 μg/mL) were pre-incubated with HAase protein (7.5 U/mL; 95 μL) in the BSA solution at 37 °C for 10 min. Then hyaluronic acid (HA; 100 μL) was added to the pre-incubated mixture at 37 °C for 45 min. HA (undegraded) was precipitated by adding an acidic BSA solution (1 mL; pH = 3.75) containing 0.1% BSA in sodium acetate (24 mM) and acetic acid (79 mM) for 10 min. The absorbance was then measured at a wavelength of 600 nm using a microplate reader (SpectraMax M2, Molecular Devices Corp., operated by SoftmaxPro v.4.6 software, Sunnyvale, CA, USA). Each sample was tested at least in three replicates. The inhibition rate was calculated using following formula: Inhibition% = 1 – [OD_HA_ – OD_sample_]/[OD_HA_ – OD_HAase_] × 100%. The IC_50_ value of each sample was determined by analysing its inhibition rates at different concentrations with a nonlinear regression algorithm by GraphPad Prism.

## Lineweaver–Burk plot

The kinetic parameters for HAase inhibition of **6c** and **6g** were obtained using a reported method with minor modifications^25^. Briefly, four concentrations of **6c** and **6g** near the IC_50_ value were selected for the inhibition assay. The HAase inhibition activity of **6c** and **6g** was measured at a series of concentrations of HA solutions (0.125–2.0 µM and 0.2–1.0 µM, respectively). The inhibition type of test compounds was determined by the Lineweaver-Burk plots.

### Circular dichroism (CD) measurement

The secondary structure of HAase protein was monitored by circular dichroism analysis with a Jasco J-720 spectropolarimeter (Jasco, Tokyo, Japan) using the following parameters: wavelength: 190 − 250 nm; bandwidth: 1 nm; path length of quartz cell: 1 mm^26^. HAase protein (0.2 mg/mL) was incubated with test samples at two concentrations (10 and 20 μg/mL) for 10 min prior to the CD measurements.

### Molecular docking

Auto Dock 4.2 software was used for the molecular docking study provided by the Rhode Island IDeA Network of Biomedical Research Excellence (RI-INBRE)^27^. The three-dimensional rigid structure of HAase was obtained from the protein data bank (PDB ID: 2PE4). The structures of OA derivatives were generated by Chemdraw 3 D (PerkinElmer Inc.; Waltham, MA, USA). All water molecules were removed and essential hydrogen atoms as well as Kollman charges were added into the HAase protein, followed by the computation of the Gasteiger charges. The amino acid residues of Gln18 and Arg20 were employed as the active sites for docking simulation to study the bind mode and interaction force. The size of the grid box was defined as 80 Å × 80 Å × 80 Å in the x, y, and z dimension with a grid spacing of 0.375 Å. The docking simulations were conducted with the Lamarckian genetic algorithm and the conformation with the lowest free binding energy was selected for analysis.

### Skin permeability

A computational method, SwissADME, was used to predict the skin permeability of OA and its derivatives using a previously reported method^28^. Briefly, the chemical structures of OA and its derivatives were imported by using their simplified molecular input line entry specification (SMILES) file generated by ChemDraw (PerkinElmer Inc.; Waltham, MA, USA). The SwissADME algorithm (http://www.swissadme.ch/) was used to obtain skin penetration related parameters including Log S_(ESOL)_, Log S_(Ali)_, and Log K_p_. Next, a biochemical based assay, namely, the parallel artificial membrane permeability assay (PAMAPA), was performed to measure the skin permeabilities of OA and its derivatives using a reported method with minor modifications^28^. The PAMPA assay kit (including Prisma HT buffers, membrane plate, reference standards) was purchased from Pion, Inc. (Billerica, MA, USA) and the assay was performed according to the manufacturer’s instructions. Test compounds and reference standards (i.e. warfarin, piroxicam, and verapamil) were prepared in DMSO stock solution (10 mM). The Prisma HT buffers were prepared and adjusted to pH at 6.5 and 7.4. In the deep-well plate, the Prisma HT buffer (1 mL) was added and mixed thoroughly and then the diluted sample (50 µM; 180 µL) was added to the donor (bottom) plate in the Pion sandwich apparatus and the Prisma HT buffer (200 µL) was added to the acceptor (top plate). The sandwich apparatus was then incubated and stirred at room temperature for 24 h. The UV absorbance of the donor and acceptor plates were recorded using a plate reader with the PION PAMPA analysis software to obtain the -LogPe values of the samples.

### Cell culture and viability

Human keratinocytes (HaCaT) cells were purchased from Sigma Aldrich Co. (St. Louis, MO, USA) and cultured in Dulbecco’s modified Eagle’s medium (DMEM; Life Technologies; Gaithersburg, MD, USA) with 10% fetal bovine serum (Gibico; Grand Island, NY, USA) at 37 °C in the presence of 5% CO_2_. The cell viability of HaCaT treated with or without OA and its derivatives was measured by the XTT assay according to manufacturer’s instructions (XTT Cell proliferation Kit II; Roche, USA). Briefly, HaCaT cells were seeded in 96-well plates at 5 × 10^3^ cells per well and allowed to attach for 12 h. Then OA and its derivatives (100 µL; 10 µg/mL) were incubated with cells for 24 h, followed by adding the XTT reagent (50 µL; 0.3 mg/mL) in each well and incubated for 4 h. The absorbance of each well was measured at wavelengths of 450 nm using a SpectraMax M2 plate reader (Molecular Devices; Sunnyvale, CA, USA).

### Cellular reactive oxygen species (ROS)

HaCaT cells were seeded in 96-well plates at 6 × 10^3^ cells per well and allowed to attach for 12 h. Then the cells were incubated with test samples (10 µg/mL) for 24 h. Next, the cell culture medium was removed and fresh phosphate buffer solution (PBS; 100 µL) containing a fluorescent probe 2′,7′-dichlorofluorescin diacetate (DCFDA; 20 µM) was added to the cells and incubated for 20 min. Then the cells were treated with H_2_O_2_ (100 µL; 200 µM) for 1 h followed by measuring the fluorescence intensity of each well with excitation and emission wavelengths of 485 and 525 nm, respectively, using a SpectraMax M2 plate reader^29^.

### Statistical analysis

Statistical analyses were performed using GraphPad Prism 8 (GraphPad Software, La Jolla, CA, USA). Data were expressed as the mean value ± standard deviation (SD). The significance of difference was analysed using a one-way analysis of variance (ANOVA) followed by a *post hoc* Student-Newman-Keuls multiple comparison test (SNK).

## Results and discussion

### Synthesis of oleanolic acid analogues

**Scheme 1.**
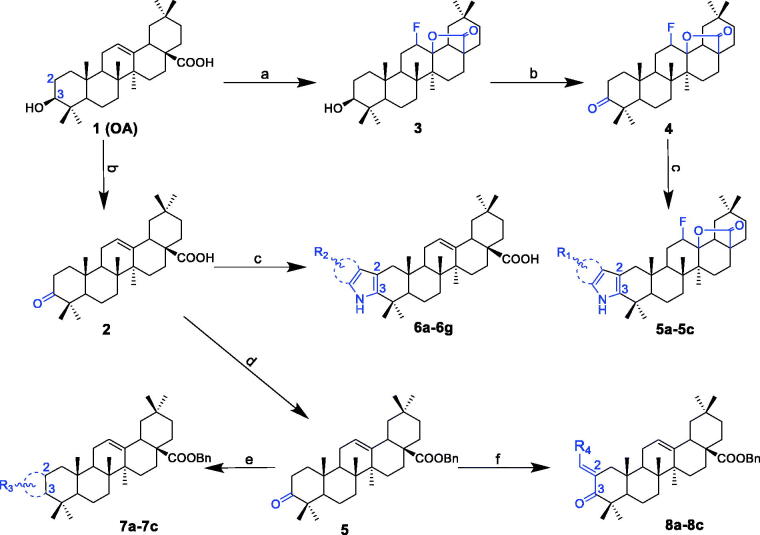
Synthesis routes of OA analogues **2**–**4**, **5a**-**5c**, **6a**-**6g**, **7a**-**7c** and **8a**-**8c**. Reagents and conditions: (a) selectflour, dioxane, nitromethane, 80 °C, 4 h; (b) the Jones reagent, acetone, at 0 °C; (c) RPhNHNH_2_, CF_3_COOH, acetic acid, 115 °C, overnight; (d) BnCl, K_2_CO_3_, r.t., overnight; (e) R-CHO (*o*-amino benzaldehyde or *o*-amino pyridylaldehyde), KOH, ethanol, refluxed, 5 h; (f) R-CHO, KOH, ethanol, r.t., overnight.

A series of OA derivatives were synthesised via structural modifications at OA’s C-2, C-3, C-28, C-12 and C-13 positions (Scheme 1). OA was first fluorinated with selectfluor (tetrafluoroborate) to obtain the intermediate analogue **3**. Another intermediate analogue **4** was obtained by modifying compound **3** with the Jones reagent. The target compounds **5a**-**5c** were prepared through condensation of compound **4** with various substituted phenylhydrazine and purified by column chromatography. Analogue **2** was synthesised by modifying OA at the C-3 position, which was oxidised to carbonyl with the Jones reagent. In addition, compounds **6a**-**6g** were synthesised by employing a variety of substituted phenylhydrazine to compound **2** using the Fischer indolization approach. Similarly, target compounds **7a**-**7c** and **8a**-**8c** were synthesised through the condensation of compound **2** with various aldehydes via the Claisen Schmidt condensation. The structures of OA analogues were confirmed by their spectroscopic data including ^1^H NMR, ^13 ^C NMR and HRMS.

### OA derivatives inhibit the activity of HAase enzyme

**Figure 1.**
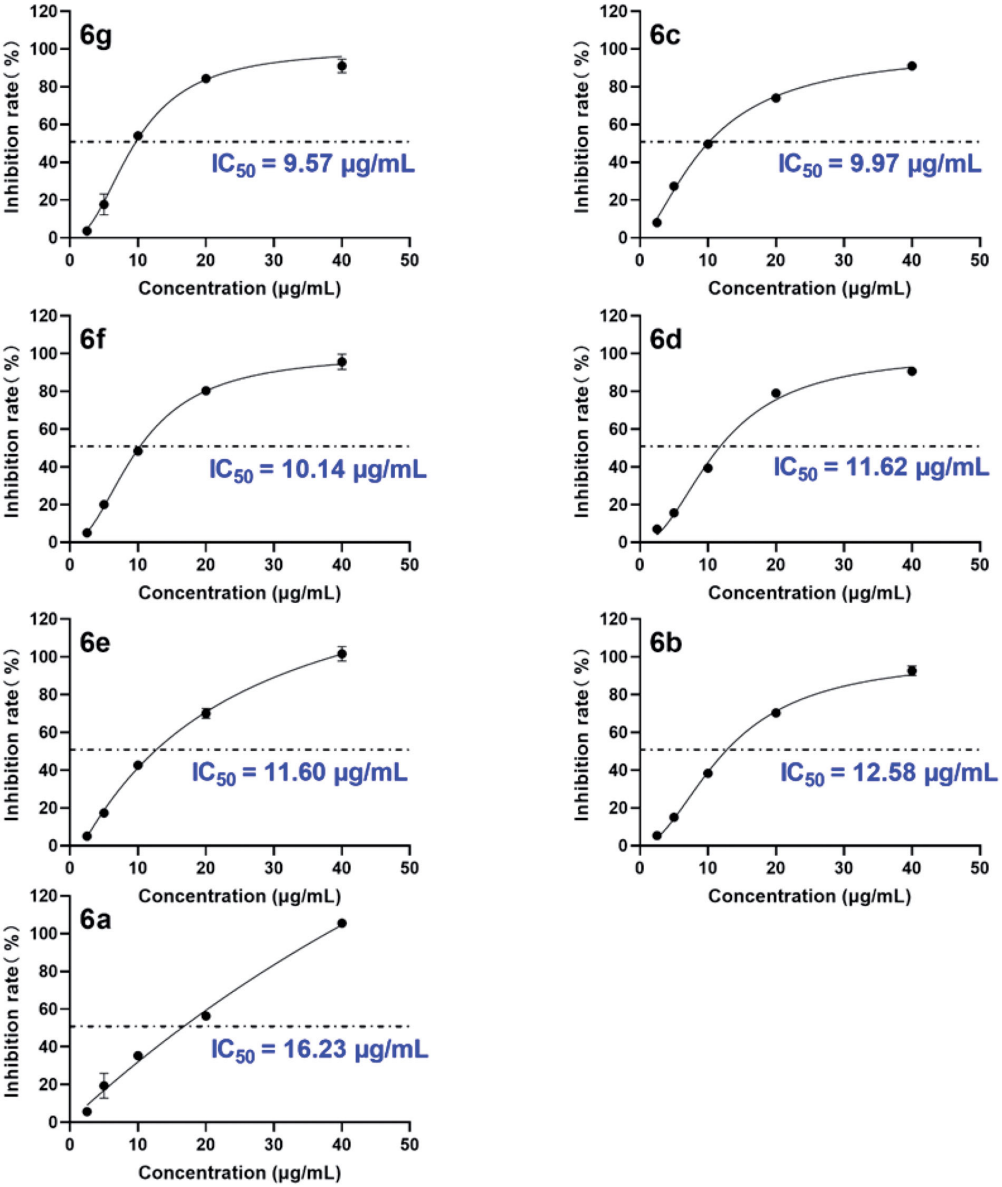
Inhibitory effects of leading compounds **6a**-**6g** on the activity of bovine testicular HAase enzyme activity. The inhibition rates (%) of each compound were measured at five concentrations (1, 5, 10, 20, and 40 µg/mL) and an inhibition curve was constructed to calculate the IC_50_ value of each test compound using nonlinear regression.

The inhibitory effects of compounds **2**-**8c** against HAase enzyme activity were evaluated. The inhibitory effect of OA analogues on HAase was first evaluated at a threshold concentration of 40 µg/mL. OA (**1**) showed a moderate inhibitory activity with an inhibition rate of 59.3% and the intermediates **2**–**4** only had weak inhibitory effects with inhibition rates less than 37.3% (Table 1). Among the OA derivatives, analogues **6a**, **6b**, **6c**, **6d, 6e**, **6f**, and **6g** bearing indole moieties showed the most potent anti-HAase effects with an inhibition rate of 100.9, 100.2, 86.3, 93.5, and 98.1%, respectively. Analogues **5a**-**5c** only showed weak inhibitory effect (18.7–37.6% inhibition, respectively). Similarly, the anti-HAase activities of OA derivatives **7a**-**7c** and **8a**-**8c** (22.6–32.5% and 20.2–25.2% inhibition rate, respectively) were weaker than analogues **6**. Next, the inhibitory effects of OA derivatives with promising enzyme inhibition activity (**6a**-**6g)** were further evaluated by obtaining their IC_50_ values. OA derivatives **6a**-**6g** had IC_50_ values ranging from 9.5–16.2 µg/mL (or 16.7–28.9 µM) (Figure 1).

**Table 1.** Inhibitory effect of **1** (OA) and its chemical synthesised derivatives **2**–**8** (at 40 μg/mL) on the enzyme activity of HAase.

Compound	MW (g/mol)	Functional group		Inhibition (%)
**1**	456.7	/		59.3 ± 2.4
**2**	454.7	/		15.9 ± 4.3
**3**	474.7	/		12.7 ± 3.9
**4**	472.7	/		37.3 ± 1.4
**5a**	629.8	R_1_=	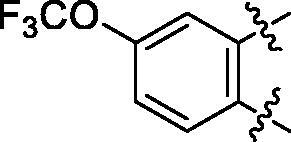	18.7 ± 3.8
**5b**	563.8	R_1_=	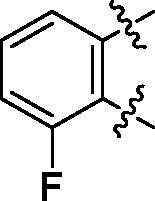	25.6 ± 3.8
**5c**	580.2	R_1_=	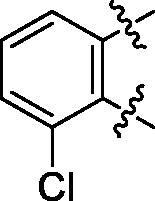	37.6 ± 4.7
**6a**	572.8	R_2_=	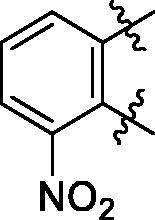	100.9 ± 4.3
**6b**	545.8	R_2_=	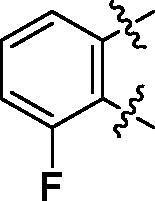	100.4 ± 2.4
**6c**	563.8	R_2_=	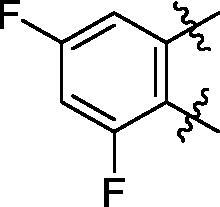	86.3 ± 3.3
**6d**	555.8	R_2_=	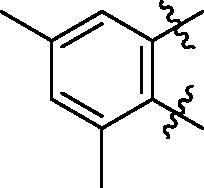	93.5 ± 3.9
**6e**	606.7	R_2_=	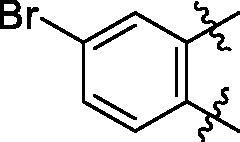	83.7 ± 3.3
**6f**	606.7	R_2_=	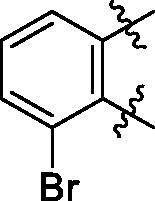	100.6 ± 3.6
**6g**	562.2	R_2_=	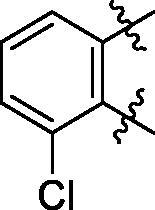	98.1 ± 0.4
**7a**	629.9	R_3_=	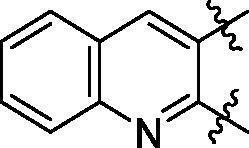	27.9 ± 4.0
**7b**	630.9	R_3_=	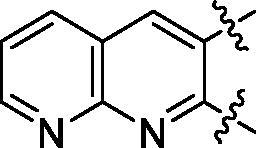	21.3 ± 3.9
**7c**	630.9	R_3_=	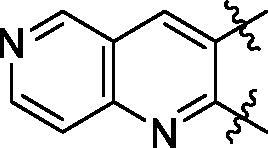	32.5 ± 1.7
**8a**	693.9	R_4_=	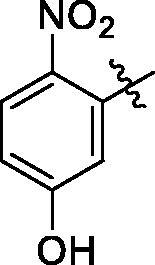	20.2 ± 2.6
**8b**	708.0	R_4_=	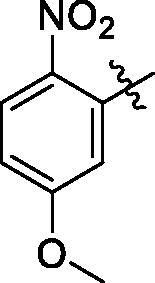	31.8 ± 0.6
**8c**	690.0	R_4_=	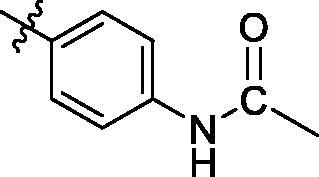	25.2 ± 4.4

Some preliminary structure and activity relationship (SAR) can be observed based on the anti-HAase activity of OA analogues bearing different functional groups at 40 µg/mL. For instance, decarboxylation at C-28 position (analogues **3**–**5**; inhibition rate of 12.7–37.6%) did not increase the anti-HAase activity as compared to OA indole derivatives with a carboxyl group at the C-28 position (analogues **6**; inhibition rate of 86.3–100.6%). In addition, OA derivatives with a carboxyl group and an indole group (analogues **6**; inhibition rate: 86.3–100.6%) showed superior inhibitory effects as compared to carbonylated OA derivatives with functional groups including pyridine ring (analogues **7**; inhibition rate: 22.6–30.1%) or benzaldehyde ring (analogues **8**; inhibition rate: 20.2–31.8%). However, there is a lack of convincing evidence to support the conclusion whether the induction of halide groups on the OA indole skeleton (**6b**, **6c**, **6e**, **6f**, and **6g**; inhibition rate: 83.7–100.6%) have superior anti-HAase activity as compared to the analogues with the same skeleton but bearing non-halide moieties **6a** and **6d** (inhibition rate: 93.5–100.9%). Further SAR studies are required to elucidate the impact of indole moieties on the OA derivatives’ anti-HAase effects. We further evaluated the mechanisms of inhibition of two leading compounds **6c** and **6g.**

### OA indole derivatives 6c and 6g interact with HAase protein

**Figure 2.**
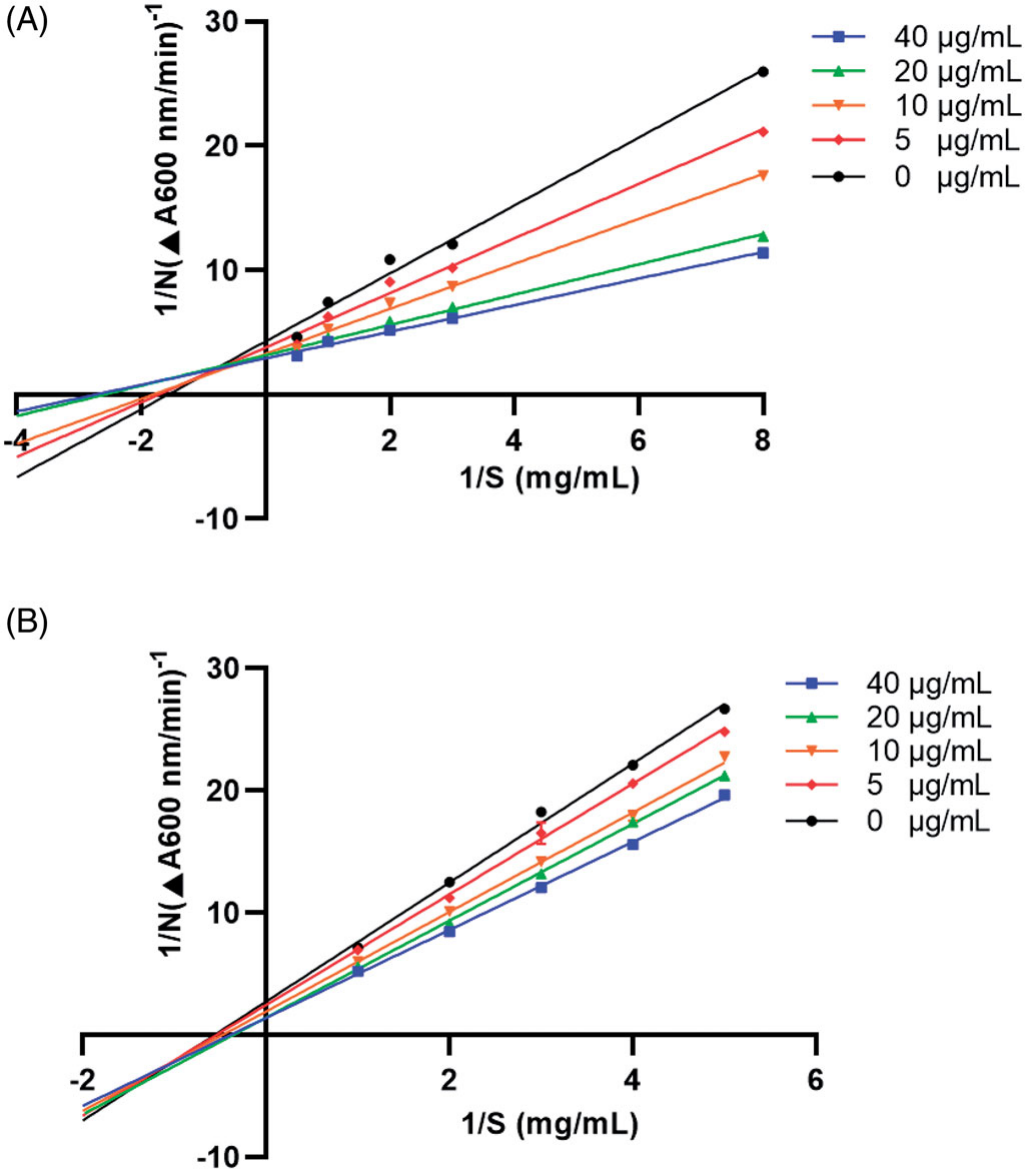
Enzyme reaction kinetics of **6c** (A) and **6g** (B) were measured by constructing the Lineweaver-Burk plots. Four concentrations (5–40 µg/mL) were co-incubated with HAase in the presence of substrate solutions (HA) at concentrations of 0.125–2.0 µM and 0.2–1.0 µM, respectively.

**Figure 3.**
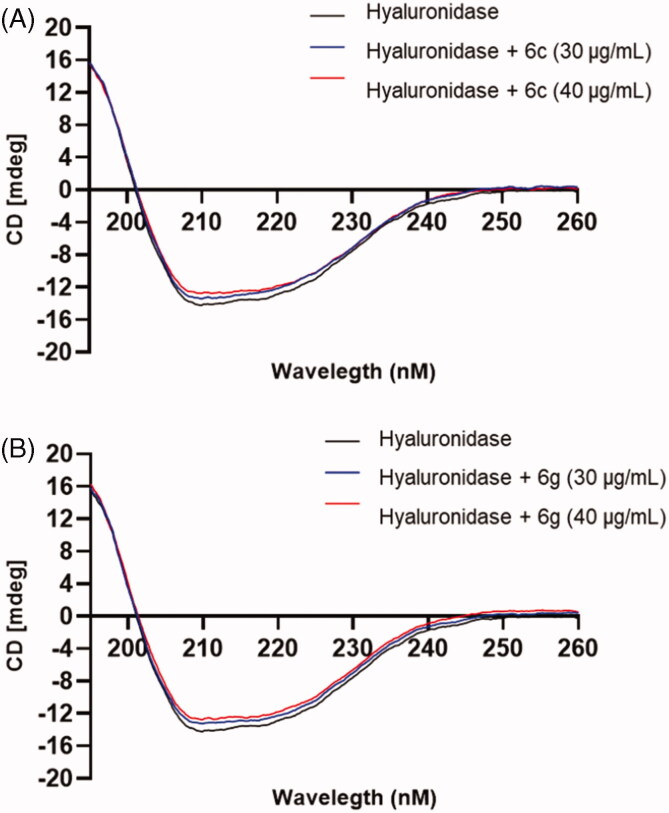
Effects of **6c** (A) and **6g** (B) on the secondary structure of HAase protein characterised by far-UV CD spectra measurements.

We next studied OA indole derivatives **6c**’ and **6g**’s mechanisms of inhibition on HAase with spectroscopic assays and computational method. First, the Lineweaver-Burk plot of **6c** or **6g** at various concentrations in the presence of substrate solutions (HA) at several concentrations was constructed, which showed straight lines on the *x*-axis at different points (Figure 2). This suggests that **6c** and **6g** had mixed type of competitive and non-competitive inhibitions on HAase. Second, the effects of HAase inhibitors (**6c** and **6g**) on the secondary structure of the enzyme protein were evaluated by the CD spectra. CD is a sensitive tool for determining the secondary structure of proteins and can be used to monitor protein’s conformational changes induced by binding interactions with small molecules^30^. The CD spectrum of intact HAase protein yielded two typical far-UV CD signals at 205 and 224 nm, which are in response to the peptide bond absorption, representing typical α-helical structures of the enzyme protein (Figure 3). Moreover, changes in the near UV region (320–260 nm) of CD spectra indicted that binding interactions occurred between **6c** or **6g** and the aromatic amino acid side chains of the enzyme protein, indicating the changes in protein’s tertiary structure. Quantitatively, co-incubation of HAase protein and **6c** or **6g** showed a reduction of the α-helix content from 35.4% to 26.1 and 29.8%, respectively. In addition, the random coil content increased from 39.3% to 42.5 and 44.7%, respectively. This suggests that HAase inhibitors disturbed the hydrogen force in the secondary structure of HAase resulting in an impaired enzyme activity. This observation is in agreement with several reported studies using CD technique to show that natural products based HAase inhibitors (for e.g. flavonoids including baicalin, liquiritin, and rutin) can bind to HAase protein and alter its secondary structures, thereby leading to undermined HAase activity^31^^,^^32^.

**Figure 4.**
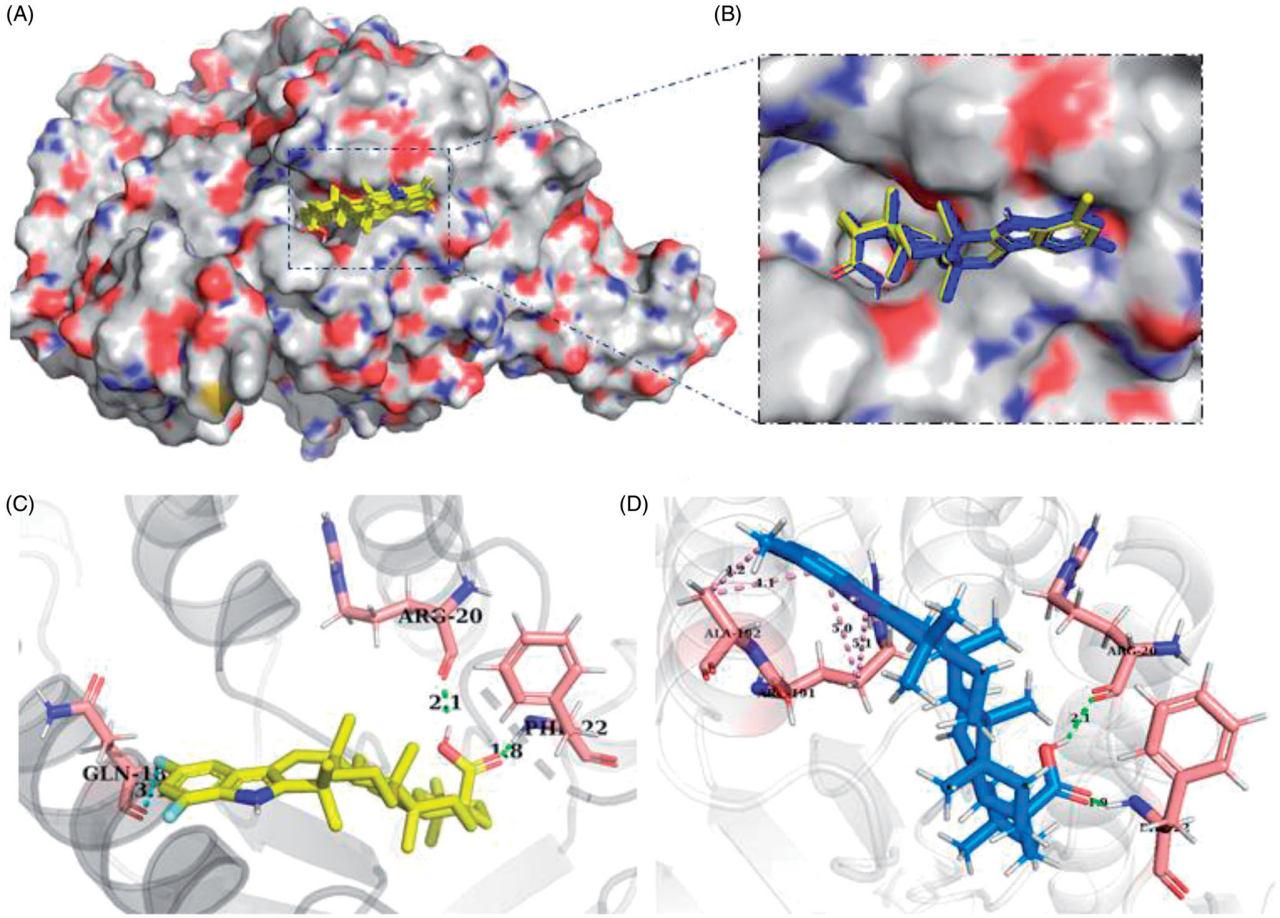
Interactions between HAase protein and OA derivatives predicted by molecule docking. OA derivatives’ potential binding pocket on HAase protein (A); Enlarged view of the binding site of leading compounds **6c** and **6 g** on HAase protein (B); The interactions between HAase protein’s amino acid residues and compound **6c** (C) and **6 g** (D).

Next, the interactions between HAase protein and OA analogues were studied by molecular docking, which predicted that OA indole derivatives including **6c** and **6g** were able to bind to HAase protein (Figure 4(A,B)). Further binding analysis of two leading HAase inhibitors **6c** and **6g** showed that they interacted with enzyme protein’s amino acid residues including Arg20, Gln18, Phi22, Arg191 and Ala192 (Figure 4(C,D)). In addition, **6c** and **6g** formed several molecular forces including hydrogen bond and alkyl-pi bond to stabilise the formation of the ligand-protein complex, which also contributed to the reduced enzyme activity. Furthermore, the free binding energy to HAase protein and the binding constant of compounds **6c**, **6d**, **6e**, **6f**, and **6g** were predicted as −9.61, −8.93, −8.15, −9.03, and −8.85 kcal/mol, and 7.85, 13.45, 15.12, 10.44, and 14.77 µM, respectively. The predicted binding parameters was in agreement with data obtained from the enzyme inhibition assay. For instance, **6c**, as one of the most active HAase inhibitor among all of the OA analogues, had the lowest free binding energy (−9.61 kcal/mol) and binding constant (7.85 µM). Data from biochemical (enzyme kinetic), biophysical (CD), and computational assays support that the protein-ligand interaction contributed to the inhibitory effect of OA derivatives on HAase enzyme activity. Apart from binding to HAase protein’s catalytic or allosteric site and altering the secondary structure of enzyme protein, other types of inhibition are involved in HAase inhibitors’ mechanisms of action. For instance, studies have shown that several factors including pH conditions and ionic strength are critical for HAase’s activity and some glycoproteins HAase inhibitors (also termed as inter-α-inhibitors) showed magnesium dependent inhibition on HAase at specific pH environment^33^. However, further studies are warranted to confirm whether OA derivatives’ inhibition HAase was mediated by aforementioned mechanism.

Several factors should be accounted for the development of HAase inhibitors based cosmeceuticals for their potential skin-ageing applications. For example, HAase inhibitors may need to penetrate the skin barrier and reach to specific skin layers (i.e. basement membrane and interstitial matrix) to exert desired biological effects. Therefore, it is critical to characterise the physico-chemical properties, such as the skin permeability, of OA derived HAase inhibitors.

### Permeability of OA and its derivatives

**Figure 5.**
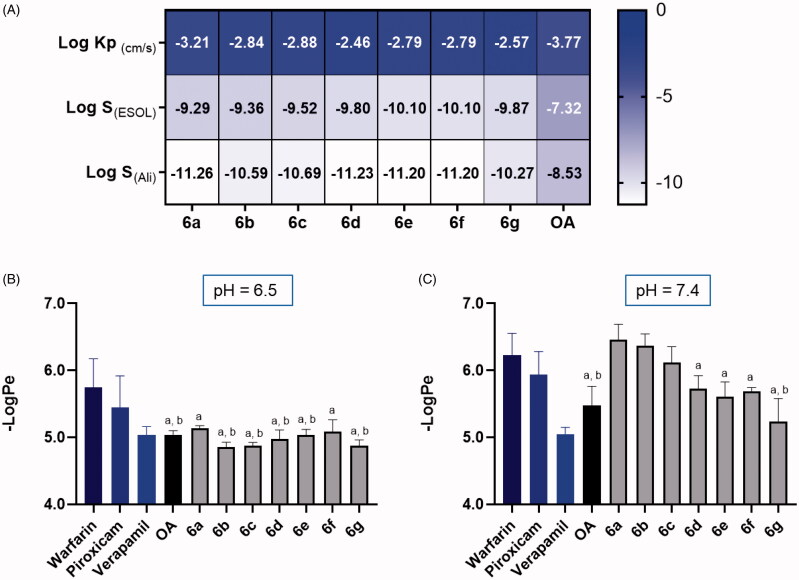
Skin permeability of OA and its derivatives (**6a**-**6g**) predicted by computational SwissADME method and obtained the PAMPA assay. Skin permeability coefficient predicted by the SwissADME (A). Skin permeability (apparent permeability; -Log Pe value) obtained by the PAMPA assay at pH = 6.5 (B) and 7.4 (C). Statistical significant difference was considered as a (*p* < 0.05) when compared to the reference compound warfarin; b (*p* < 0.05) when compared to the reference compound piroxicam; c (*p* < 0.05) when compared to the reference compound verapamil.

The skin permeability of OA and its derivatives was assessed by in silico and *in vitro* models. First, the skin permeability coefficient (Log K_p_; cm/s) of OA and its derivatives (**6a**-**6g**) was predicted by the SwissADME algorithm based on their physico-chemical properties including molecular weight, lipophilicity, and Log P_o/w_. The more negative the coefficient value is, the less possible for the compound to penetrate the skin barrier. The SwissADME method predicted that OA and its derivatives have feasible skin permeability with Log K_p_ values of −3.77 and −3.21 to −2.46 cm/s, respectively (Figure 5(A)). Notably, structural modified OA derivatives showed superior skin permeability as compared to OA. This observation was further supported by OA derivatives’ apparent permeability (Log Pe) value obtained from the PAMPA assay at pH conditions of 6.5 and 7.4. Compounds **6a**-**6g** had -Log Pe values of of 5.13, 4.86, 4.88, 4.98, 5.03, 5.09, and 4.87, respectively, in a slightly acidic condition (pH = 6.5; Figure 5(B)). The PAMPA assay reference standards including warfarin, piroxicam, and verapamil, respectively representing low, medium, and high skin permeability, had a -Log Pe value of 5.75, 5.44, and 5.04, respectively, at pH = 6.5. This suggest that the skin permeability of several OA derivatives including **6b**, **6c**, and **6g** was enhanced at pH = 6.5 as compared to OA (4.86–4.88 v.s. 5.04), which supported the prediction of the skin permeability of OA derivatives in the SwissADME model. However, OA’ and its derivatives’ (**6a**-**6g**) -Log Pe value significantly increased to 5.3 and 6.46, 6.37, 6.12, 5.73, 5.61, 5.69, and 5.24, respectively, when the pH condition adjusted to 7.4 (Figure 5(C)), suggesting that the skin permeability of OA derivatives may be undermined in a slightly basic environment.

Data obtained from the PAMPA assays provided useful insights for the further development of OA indole derivatives as cosmeceuticals. For instance, OA derivatives are more skin permeable in a slightly acidic condition (pH = 6.5). This might be favourable in healthy skin conditions given that the skin’s surface and uppermost layers (for e.g. stratum corneum) are acidic^34^. In addition, the acidic pH is preferrable for a variety of cosmetic products, such as cleansers and toners (pH 4.5–6), serums (pH 4–6), vitamin C (ascorbic acid) products (pH 2.6–3.2) and retinol products (pH 4.0–6.6)^35^. However, there is a limitation in our current study. The skin permeabilities of the OA derivatives were assessed using computational method (the SwissADME prediction) and membrane based *in vitro* model (the PAMPA assay). Results obtained from these *in silico* and *in vitro* assays may not accurately reflect OA derivatives’ skin permeability. Skin is the largest organ with perhaps the most complex biological functions in the body. One of the major physiological purposes of skin is to protect the body from external substances including pathogen, UV radiation, and free radicals^36^^,^^37^. This protection is enabled by the involvement of numerous skin cells including dermis (which is consisting of fibroblasts, sweat glands, dermal adipose cells, mast cells, and infiltrating leucocytes) and epidermis (which is constituted by keratinocytes, melanocytes, Langerhans cells, and Merkel cells)^38^^,^^39^. Altogether, these cells form four distinct layers including the stratum basale, stratum spinosum, stratum granulosum, and stratum corneum, which determines to the rigidity of skin permeability^39^^,^^40^. Therefore, further evaluations of the skin permeability of OA derivatives using in vivo models are warranted.

### OA derivative 6c reduces H_2_O_2_-induced cellular reactive oxygen species (ROS) in HaCaT cells

**Figure 6.**
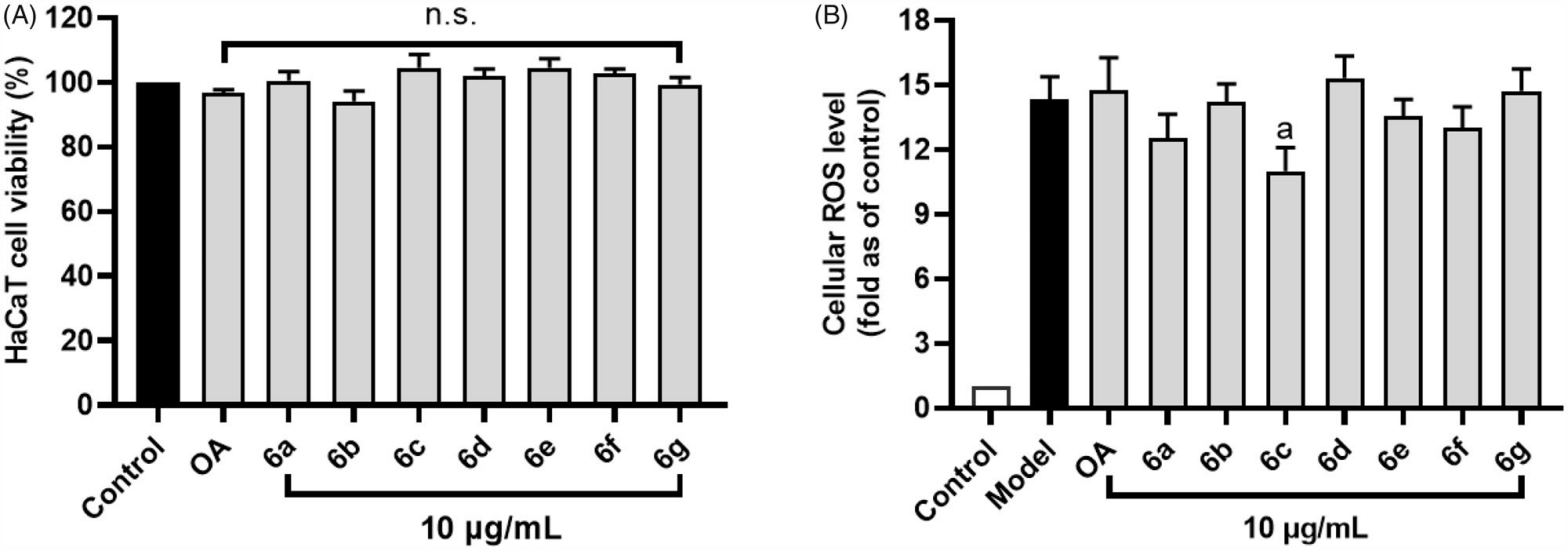
Effects of OA and its derivatives on the cell viability of human keratinocyte HaCaT cells (A) and their antioxidant effect against H_2_O_2_-induced production of ROS in HaCaT cells (B). Data are presented as means ± SD from experiments with three replicates. Statistical significant difference was considered as a (*p* < 0.01) when compared to the model (H_2_O_2_-exposed) group. n.s. = not significant.

The potential skin beneficial effects of OA and its derivatives were evaluated by cellular assays. First, cytotoxicity of OA and its derivatives was evaluated in human keratinocytes HaCaT cells. Treatment with OA and its derivatives **6a**-**6g** at a concentration of 10 µg/mL resulted in a cell viability of 96.68 and 94.13–104.55%, respectively, suggesting that these compounds are non-toxic to HaCaT cells at the tested concentration (Figure 6(A)). In addition, the skin protective effect of OA derivatives was evaluated against H_2_O_2_-induced oxidative stress by assessing the level of ROS generated in HaCaT cells. The ROS level in H_2_O_2_-stimulated cells was elevated by 13-fold as compared to the control group. Treatment with compound **6c** (10 µg/mL) showed antioxidant effect on HaCaT cells by decreasing the ROS level by 23.6% as compared to the control group (H_2_O_2_-exposed cells) (Figure 6(B)). Other OA analogues did not significantly ameliorate H_2_O_2_-induced oxidative stress.

Apart from the anti-HAase activity, OA also shows inhibitory effects on the activity of skin-ageing related enzymes including elastase^41^ and collagenase^42^, which may contribute to the overall anti-skin-ageing activity of OA. Similar to the anti-HAase activity, it is possible that the OA indole derivatives may exert enhanced anti-elastase and anti-collagenase effects but further enzymatic assays are warranted to confirm this. In addition, it was reported that OA inhibits particulate matter induced release of matrix metalloproteinase 1 (as known as interstitial collagenase) in dermal fibroblasts^3^. The current study also showed that OA inhibits particulate matter induced skin ageing which results in the release of pro-inflammatory cytokines including tumour necrosis factor-alpha and interleukin 6. Thus, OA’s anti-inflammatory effects may also have contributed to the overall skin protective effects against skin ageing. Moreover, an in vivo study using a mouse model showed the skin protective of OA against toxin (12-O-teradecanoyl-phorbol-13-acetate) induced skin carcinogenesis via the inhibition of aberrant gene expressions^43^. Therefore, further studies on the evaluations of OA indole derivatives’ skin beneficial effects using in vivo models are warranted.

## Conclusion

In summary, a series of OA analogues were structurally modified by the induction of indole moieties via the Fischer indole synthesis. Several OA indole derivatives bearing halogen atom(s) showed superior anti-HAase activity as compared to the parent compound OA. The mechanistic studies using biochemical, biophysical, and computational assays suggest that the inhibitory effects of OA derivatives on HAase may be associated with their binding capacity to HAase protein. Moreover, OA derivatives showed feasible skin permeability from in silico (SwissADME) and *in vitro* (PAMPA) assays. Further biological evaluations of potential skin protective effects of OA derived HAase inhibitors in human keratinocytes showed that OA analogue with enhanced skin permeability also exerted cellular antioxidant effects. Findings from the current study provide useful insight on the development of OA based HAase inhibitors as cosmeceutical agents but further investigations are warranted to assess their anti-skin-ageing effects using in vivo models.
